# Pilot-drill guided vs. full-guided implant insertion in artificial mandibles—a prospective laboratory study in fifth-year dental students

**DOI:** 10.1186/s40729-019-0176-4

**Published:** 2019-06-26

**Authors:** Matthias C. Schulz, Francisca Hofmann, Ursula Range, Günter Lauer, Dominik Haim

**Affiliations:** 1 Department of Oral and Maxillofacial Surgery, Faculty of Medicine “Carl Gustav Carus”, Technische Universität Dresden, Fetscherstraße 74, 01307 Dresden, Germany; 2 Institute for Medical Informatics and Biometry, Faculty of Medicine “Carl Gustav Carus”, Technische Universität Dresden, Blasewitzer Str. 86, 01309 Dresden, Germany

**Keywords:** Full-guided implant insertion, Laboratory study, Orientation template

## Abstract

**Background:**

As a growing field in dentistry, the practical education during the undergraduate curriculum in implant dentistry should be extended. Not only the theoretical background but also practical skills are crucial to place implants in patients. In order to determine the exact implant position, several positioning aids are available. In the present laboratory study, the accuracy of implant insertion using two different guiding modes in a group of inexperienced participants was assessed.

**Methods:**

After three-dimensional planning using the data of a cone beam computed tomography of artificial mandibles, surgical templates were manufactured by thermoforming. In region 35, a sleeve for the pilot drill was used, whereas in region 45, a sleeve allowing a full-guided implant insertion was inserted. Subsequently, a total of 104 implants were placed by 52 undergraduates. Radiographical assessment of the three-dimensional accuracy was performed. Furthermore, the time required to insert the implants was recorded. Statistical analysis followed.

**Discussion:**

When comparing the three-dimensional accuracy of the virtually planned to the actual inserted implant, a statistically significantly higher accuracy in three-dimensional angulation was achieved for the full-guided (3.388 ± 1.647°) compared to the pilot-drill guided mode (5.792 ± 3.290°). Furthermore, the time required to insert the implant was shorter for the full-guided template (6.23 ± 1.78 min) vs. for the pilot-drill guided (8.84 ± 2.39 min). Both differences reached a statistical significance (*p* < 0.001).

**Conclusion:**

Within the limit of this laboratory study, the results suggest that inexperienced surgeons benefit from a full-guided implant insertion. However, the clinical effects have to be discussed as the mismatch was varying in the decimillimeter range.

## Background

Over the recent years, implant dentistry has developed to an emerging therapy in the treatment of partial or fully edentulous patients. Nowadays, the treatment with implant-borne single crowns, bridges, and removable dentures is an established option for many patients. Besides performing the correct surgical protocol properly, the determination of the correct position of the implant with respect to anatomical structures such as the maxillary sinus, inferior alveolar nerve, and adjacent teeth is considered to be a crucial factor for long-term implant success [1]. Variable methods are available to determine the exact position of the implant ranging from free-hand positioning, simply guiding the pilot drill, guided preparation of the implant cavity, to full-guided insertion of the implant [2]. It has been demonstrated that a three-dimensional planning and guiding of the implant cavity preparation resulted in a higher accuracy compared with free-hand implant insertion and that even experienced surgeons would benefit from three-dimensional guidance [3–6]. However, inexperienced surgeons might benefit more from guidance by a template [7].

It seems it would be helpful to provide undergraduate students with not only a theoretical background but also practical training under close guidance when performing their first steps in dental implantology [8, 9]. The placement of dental implants is a surgical procedure and thus carries potential risks and complications such as injuring of the inferior alveolar nerve or damage of the roots of adjacent teeth. Hence, it is necessary to train undergraduates using models before inserting dental implants in patients. It has been stated that the practical training and a familiarity with the specific surgical protocols are essential for dental students before placing dental implants clinically [10, 11]. Dimitrijevic et al. reported that in a skill test, the estimation of correct distances and depths was a problem for the majority of dental undergraduates [12]. This might cause problems when having to determine the correct position of a dental implant. In order to provide undergraduate students a possibility to train these skills in vitro and become familiar with a commonly applied method of determining the correct implant position and angulation, a laboratory implant course where the planned implant positions are transferred by the use of a template was established in the dental curriculum in our faculty. The use of templates enabled the instructors to give students feedback on their achieved accuracy. This feedback might be considered as a kind of quality-controlled training which can be directly related to the patients’ safety [13].

The aim of this study was to assess the accuracy of implant insertions by an undergraduate population using templates made by thermoforming enabling (a) a full-guided implant insertion and (b) the guidance of the pilot drill only. It was hypothesized that the implant insertion in a full-guided mode would be more accurate compared with the guided pilot drill mode and would allow a faster implant insertion. Therefore, a group of fifth-year dental students was recruited, and additional to the assessment of accuracy, individual demographic factors of age, sex, handedness, and a potential professional education prior to starting dental school were recorded.

## Methods

The study was approved by the local ethical review board (IRB00001473; file reference: EK507122017). The study was performed in accordance with the Declaration of Helsinki. All participants gave their written informed consent before taking part.

**Fig. 1:**
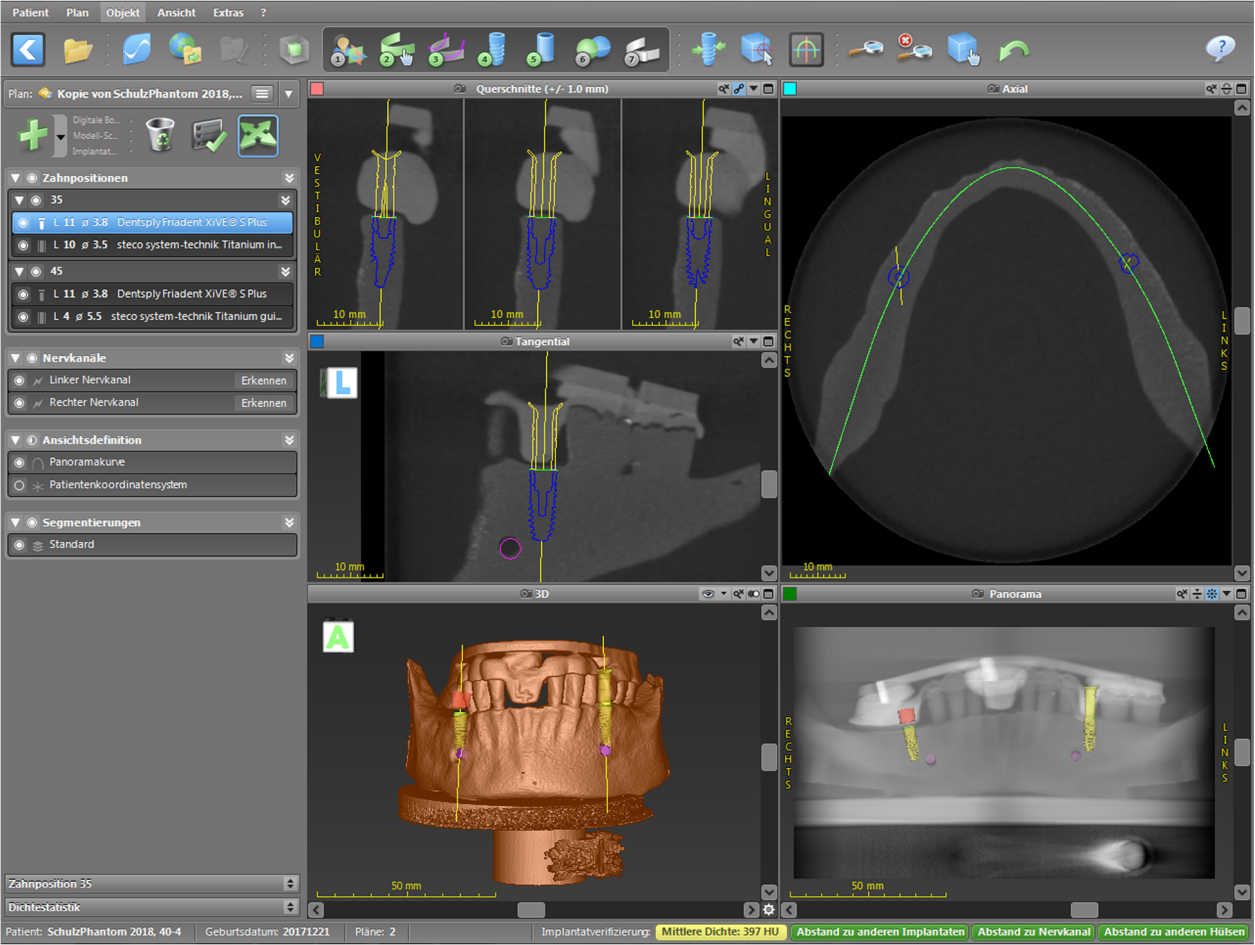
Virtual planning of the implant position and angulation in region 35 and 45 using the coDiagnostiX^TM^ software. Images in clockwise order: upper right—horizontal plane. The green elliptical line depicts the panoramic curve. Lower right—panoramic view of the mandibular model with virtually inserted implants in region 35 and 45. Lower left—three-dimensional reconstruction of the mandibular model. Center left—lateral aspect of the virtually planned implant in region 35. The pink circle located anterior-caudal of the implant depicts the mental foramen. Upper left—anterior aspect of the virtually planned implant in region 35

Artificial partially edentulous mandibular models resembling bone quality D2 (Mandibula Typ A, GOS® GmbH, Northeim, Germany) according to Misch were used [14]. First, an alginate impression was taken and a plaster cast (type IV; Excalibur, Dr. Böhme & Schöps Dental GmbH, Goslar, Germany) of the model was manufactured. X-ray splints were fabricated on those plaster casts by thermoforming using a plastic sheet (Erkodur 1.5 mm; Erkodent GmbH, Pfalzgrafenweiler, Germany) attached to a refFIX™ disk which contained three titanium pins (RefPin, IVS Solution AG, Chemnitz, Germany). Next, a cone-beam computed tomography (CBCT, Accuitomo, J. Morita Corporation, Osaka, Japan) of the X-ray splint fixed on the mandibular model was carried out. The parameters were set up according to a previous study [15]: tube voltage, 60.0 kV; current, 3 mA; exposure time, 17.5 s; gantry angle, 0.0°; field of view, 80 × 80 × 80 mm; voxel size, 0.160 mm. All data were transformed in the Digital Imaging and Communications in Medicine (DICOM) format and were transferred to the planning software (coDiagnostiX™, Dental Wings, Chemnitz, Germany). The planning of the implant positions was performed according to clinical guidelines taking into account bone height and width, distance and angulation to the adjacent teeth, and the distance to the mental foramen (Fig. 1). On the basis of the planned implant positions, four templates were manufactured using the gonyX® device (Institute Straumann AG, Basel, Switzerland). In region 35, where the implant insertion was planned to be performed with an orientation template (half-guided), two different sleeves were inserted in the template. In order to assess the influence of the length of the orientation sleeve, either a long sleeve (length 10 mm, inner diameter 2.35 mm; steco-system-technik GmbH & Co. KG, Hamburg, Germany) or a short sleeve (length 4 mm, inner diameter 2 mm; 3D Diagnostix Incorporation, Boston, Massachusetts, USA) were inserted. In region 45, a sleeve for a full-guided implant insertion with an outer diameter of 5.5 mm and a length of 4 mm (titanium guided Z; steco-system-technik GmbH & Co. KG, Hamburg, Germany) was inserted into the template.

Two identical dummy implants according to the Xive® S Plus system (Dentsply Sirona Implants, Mannheim, Germany) were inserted in the artificial mandibles in region of tooth 35 (guided pilot drill) and region of tooth 45 (fully guided). The implants had a diameter of 3.8 mm and a length of 11 mm.

The implant cavities were prepared without irrigation following the protocol of the manufacturer. The Frios Unit S/i (W&H Dentalwerk Bürmoos GmbH, Bürmoos, Austria) was used as surgical motor. For the half-guided implant insertion, the Xive® surgical tray was used, and for the full-guided implant insertion, the Xive® GS tray with drill sleeves for each bur was used (both Dentsply Sirona Implants, Mannheim, Germany). Following the insertion of the implants, the temporary abutment was removed, and the appropriate cover screw was inserted. Additionally, the time required to prepare the implant cavity and to insert the implant was recorded. Next, the artificial mandibles with the appropriate template attached to the model were scanned in the CBCT applying the same parameters in the pre-operative scans.

**Fig. 2:**
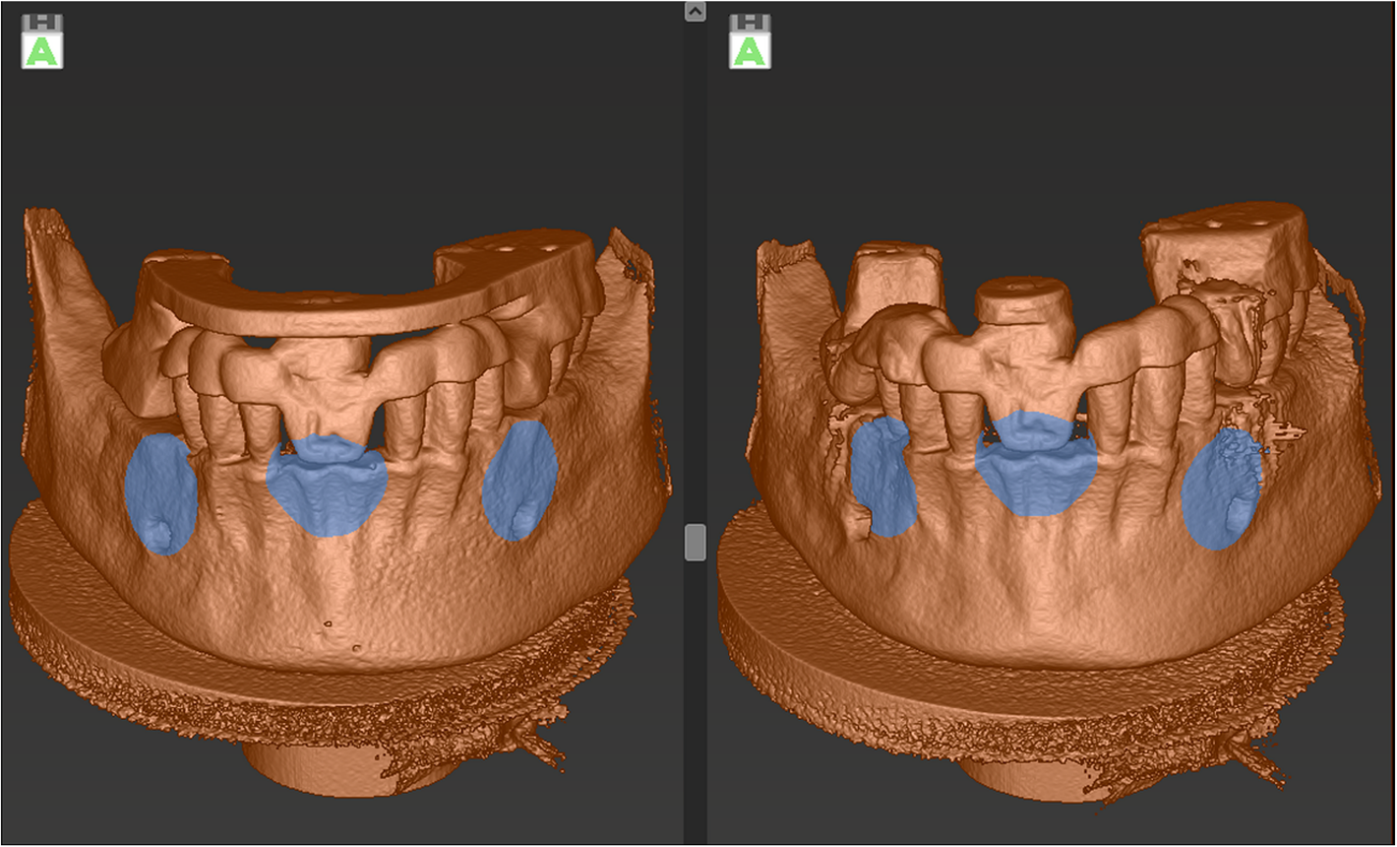
Superimposition of the pre-operative scan (left image) and the post-operative scan of the mandibular model with the attached template. The gray circular areas are depicting the region in which both scans are matched to each other

**Fig. 3:**
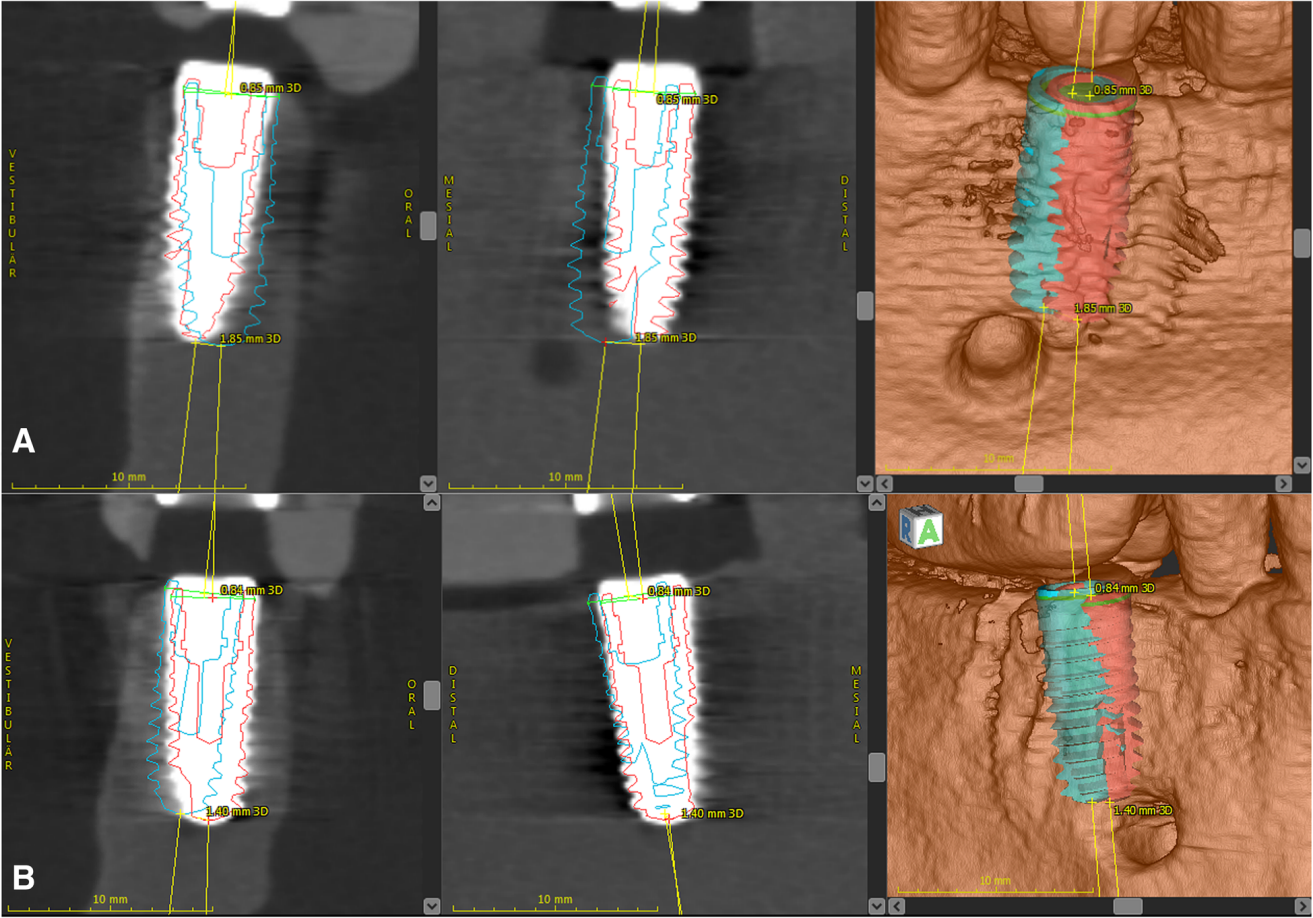
Three-dimensional superimposition of the pre-operatively planned position (blue) and the actual position (red) of the implant. **a** In this case, a mesio-lingual mismatch of the implant in region 35 is detectable. **b** In region 45, there is a slight mismatch to the distal and vestibular direction. In both images, the implant tip shows a higher deviation than the implant base

In order to evaluate the mismatch and deviation between the inserted implant and the planned position, the i-Dixel OneVolumeViewer 2.6.0. software (J. Morita MFG. CORPORATION, Kyoto, Japan) was used as described previously [15]. The implant region was analyzed in the axial, sagittal, and coronal plane applying a zoom level of 400%. The angle between the longitudinal median axis of the sleeve and the longitudinal median axis of the implant was measured to assess the deviation in bucco-lingual and mesio-distal direction. Additionally, the mismatch between the median longitudinal axis of the sleeve and the implant at the artificial bone level was measured in both these directions. The three-dimensional position of the inserted implant compared with the planned position was assessed using the “treatment evaluation” tool of the coDiagnostiX™ software. First, the pre-operative and the post-operative three-dimensional scan of the mandibular model were matched by matching three congruent parts of each scan (Fig. 2) a precise adjustment performed manually by the examiner. Then, the three-dimensional mismatch between the planned and the inserted implant was calculated by the software (Fig. 3). In order to determine the direction of the deviation and mismatch, different algebraic signs were applied: when the direction was mesial, oral, or cranial, a negative sign was used; when the direction was distal, buccal, or caudal, a positive sign was used. The recorded values were collected in an Excel® chart (Microsoft Inc., Redmond, Washington, USA).

### Statistical analysis

A statistical analysis was carried out with SPSS Statistic 25 (IBM Corporation, Armonk, New York, USA). The mean values and their standard deviations as well as the median values were computed. The Shapiro-Wilk and Kolmogorov-Smirnoff tests were used to test for a normal distribution. For the comparison of the implant insertion mode, the Mann-Whitney test was applied, and for the comparison of the templates, the Kruskal-Wallis test was applied. Statistical significance was set at *α* = 0.05. For the exact statistical significance, a Bonferroni adjustment was performed.

## Results

### Demographic data

In total, 52 participants (34 female, 18 male) completed the study and each placed two implants. The mean age was 25.8 years with a range from 22.1 to 37.3 years. One female participant was left-handed. The other participants were right-handed. Prior to joining dental school, eight students had completed a professional education. Five had graduated as dental assistants, two as healthcare assistants, and one each in the field of hotel business and piano manufacturing. All demographic data are shown in Table 1.

**Table 1:** Demographical data of the participants giving an overview about the distribution of age, sex, handedness and potential professional education

	Group	Dental students
Parameter		
Age	< 25 years	54
	> 25 years	7
Gender	female	38
	male	14
Handedness	left	1
	right	51
Professional training before dental school	no	43
	yes	9
	- dental assistant	5
	- healthcare assistant	2
	- piano maker	1
	- hotel business	1

### Deviation and mismatch between sleeve and inserted implant

The deviations of the longitudinal axis of the sleeve and the implant were higher in the pilot-drill guided implant insertion, which displayed an obvious deviation to the mesial and buccal direction. In contrast, for the full-guided implant insertion, the deviation was observed to the distal and buccal direction. When comparing the mismatch between the sleeve and the inserted implant at the implant base, a slight mesial and buccal mismatch was found for both insertion modes. For the mesio-distal direction, this mismatch was higher for the full-guided than for the pilot-drill guided implant insertion. In this case, the difference was statistically significant (*p* < 0.001). For the other values, no statistically significant differences could be found. The detailed data are depicted in Table 2.

**Table 2:** Comparison of deviations and angulations between pilot-drill guided and full-guided implant insertion. The means, their standard deviations (SD), medians, minima and maxima are shown. The p-values are resulting from a pair-wise comparison. P-values for statistically significant differences are marked in bold font

Parameter	Group	Mean	± SD	Median	Minimum	Maximum	p-value
Mesio-distal deviation implant/sleeve	pilot-drill guided	-3.85	5.35	-2.89	-18.00	6.28	0.144
In degrees	full-guided	0.41	2.65	0.63	-5.50	6.60	
Oro-vestibular deviation implant/sleeve	pilot-drill guided	0.72	0.85	0.85	-8.75	5.25	0.332
In degrees	full-guided	0.31	2.69	0.28	-5.70	7.60	
Mesio-distal mismatch implant/sleeve	pilot-drill guided	-0.02	0.38	0.00	-0.77	0.84	**< 0.001**
In mm	full-guided	-0.14	0.39	-0.10	-1.13	0.90	
Oro-vestibular mismatch implant/sleeve	pilot-drill guided	0.24	0.43	0.25	-0.90	1.00	0.079
In mm	full-guided	0.15	0.47	0.17	-1.50	1.15	
Three-dimensional angulation	pilot-drill guided	5.792	3.290	4.850	0.300	13.600	**< 0.001**
In degrees	full-guided	3.388	1.647	3.300	0.300	7.500	
Mesio-distal mismatch at implant base	pilot-drill guided	0.011	0.527	0.040	-0.980	0.970	0.601
In mm	full-guided	-0.027	0.437	-0.025	-0.910	1.060	
Oro-vestibular mismatch at implant base	pilot-drill guided	0.048	0.575	-0.005	-1.090	1.390	0.284
In mm	full-guided	0.129	0.562	0.160	-1.380	1.380	
Vertical mismatch at implant base	pilot-drill guided	-0.304	0.647	-0.325	-2.150	1.220	**0.026**
In mm	full-guided	-0.040	0.448	-0.005	-0.910	1.220	
Cumulated mismatch at implant base	pilot-drill guided	0.962	0.424	0.925	0.090	2.380	**0.007**
In mm	full-guided	0.768	0.355	0.700	0.170	2.060	
Mesio-distal mismatch at implant tip	pilot-drill guided	0.358	1.184	0.085	-1.770	3.010	0.231
In mm	full-guided	0.055	0.936	0.040	-1.560	2.100	
Oro-vestibular mismatch at implant tip	pilot-drill guided	-0.459	1.033	-0.525	-2.670	1.980	0.308
In mm	full-guided	-0.274	0.949	-0.225	-2.180	2.240	
Vertical mismatch at implant tip	pilot-drill guided	-0.235	0.645	-0.180	-2.120	1.250	**0.047**
In mm	full-guided	0.001	0.453	0.010	-0.890	1.240	
Cumulated mismatch at implant tip	pilot-drill guided	1.662	0.685	1.660	0.260	3.030	**0.004**
In mm	full-guided	1.304	0.574	1.185	0.450	2.750	

When inspecting the models visually, twelve perforations of the buccal (eight) or the lingual (four) wall were obvious. Seven occurred when using the full-guided mode and five when using the guided pilot drill. No statistically significant difference could be found between both insertion modes when tested using the Pearson chi-square test (*p* = 0.720).

### Differences between virtually planned and really inserted implant

When comparing the three-dimensional position of the planned implant and the inserted implant using the “treatment evaluation” tool of coDiagnostiX™, it was obvious that the three-dimensional angulation achieved with the full-guided implant insertion was closer to the planned position than in the guided pilot drill mode. This difference was statistically significant (*p* < 0.001). Interestingly, the mismatch in the mesio-distal and bucco-lingual direction measured at the implant shoulder was slightly higher for the full-guided implant insertion when compared with the pilot-drill guided mode. However, no statistically significant difference could be found (*p* = 0.601; *p* = 0.284). In contrast, the mismatch in the vertical direction was statistically significantly higher for the pilot-drill guided implant insertion (*p* = 0.026). Thus, the cumulated three-dimensional mismatch was higher for the pilot-drill guided mode and reached a statistically significant difference between both implant insertion modes (*p* = 0.007). At the tip of the implant, the mismatch between the planned and the inserted implant was lower for the full-guided implant insertion in all directions. Again, a statistically significant difference was reached for the vertical and the three-dimensional mismatch (*p* = 0.047; *p* = 0.004).

### Discrepancy between long and short sleeves

When the guided pilot drill implant insertion was performed using the long sleeves, for most parameters, a higher accuracy was achieved. However, statistical significance was reached only for mismatch in the mesio-distal direction and for the three-dimensional angle (*p* = 0.035; *p* = 0.007 respectively). For the mesio-distal mismatch at the implant base, values for both sleeves were comparable. The cranio-caudal mismatch was lower when using the short sleeves. Thus, the three-dimensional mismatch assessed at the implant base and tip was higher for the group with the long sleeves. No statistically significant differences could be observed (Table 3).

**Table 3:** Comparison of deviations and angulations between short sleeves (4 mm) and long sleeves (10 mm) for the pilot-drill guided implant insertion. The means, their standard deviations (SD), medians, minima and maxima are shown. The p-values are resulting from a pair-wise comparison. P-values for statistically significant differences are marked in bold font

Parameter	Group	Mean	± SD	Median	Minimum	Maximum	p-value
Mesio-distal deviation implant/sleeve	short sleeve	-5.368	6.640	-2.900	-18.000	6.280	0.315
In degrees	long sleeve	-2.637	3.748	-2.870	-10.500	5.800	
Oro-vestibular deviation implant/sleeve	short sleeve	0.995	2.068	0.800	-3.850	5.250	0.832
In degrees	long sleeve	0.498	2.968	0.900	-8.750	4.000	
Mesio-distal mismatch implant/sleeve	short sleeve	-0.154	0.395	-0.200	-0.750	0.550	**0.035**
In mm	long sleeve	0.080	0.333	0.130	-0.770	0.840	
Oro-vestibular mismatch implant/sleeve	short sleeve	0.317	0.522	0.400	-0.900	1.000	0.103
In mm	long sleeve	0.184	0.341	0.120	-0.360	1.000	
Three-dimensional angulation	short sleeve	7.357	3.729	7.200	1.400	13.600	**0.007**
In degrees	long sleeve	4.552	2.279	4.400	0.300	10.600	
Mesio-distal mismatch at implant base	short sleeve	-0.043	0.555	-0.020	-0.960	0.860	0.549
In mm	long sleeve	0.053	0.510	0.060	-0.980	0.970	
Oro-vestibular mismatch at implant base	short sleeve	0.118	0.655	0.060	-0.920	1.370	0.519
In mm	long sleeve	-0.009	0.509	-0.030	-1.090	1.390	
Vertical mismatch at implant base	short sleeve	-0.253	0.368	-0.260	-0.980	0.340	0.685
In mm	long sleeve	-0.345	0.807	-0.390	-2.150	1.220	
Cumulated mismatch at implant base	short sleeve	0.897	0.335	0.920	0.170	1.640	0.402
In mm	long sleeve	1.013	0.482	0.930	0.090	2.380	
Mesio-distal mismatch at implant tip	short sleeve	0.417	1.515	-0.060	-1.770	3.010	0.919
In mm	long sleeve	0.310	0.853	0.200	-1.280	1.800	
Oro-vestibular mismatch at implant tip	short sleeve	-0.664	1.074	-0.850	-2.670	1.850	0.242
In mm	long sleeve	-0.297	0.988	-0.500	-1.800	1.980	
Vertical mismatch at implant tip	short sleeve	-0.150	0.352	-0.150	-0.960	0.440	0.362
In mm	long sleeve	-0.302	0.806	-0.350	-2.120	1.250	
Cumulated mismatch at implant tip	short sleeve	1.880	0.737	1.850	0.510	3.030	0.070
In mm	long sleeve	1.488	0.598	1.460	0.260	2.460	

### Required time for implant insertion

The mean time for the guided pilot drill implant insertion was 8.84 ± 2.39 min. For the full-guided insertion mode, the mean time was 6.23 ± 1.78 min. When comparing both modes considering the required time for implant cavity preparation and implant insertion, a statistically significant difference could be found (*p* < 0.001). Interestingly, when using the short sleeves for the guided pilot drill implant insertion, the participants required more time compared with the long sleeves. This difference was statistically significant (*p* = 0.041). The detailed data are depicted in Table 4.

**Table 4:** Comparison of the time required for implant insertion for pilot-drill guided and full-guided implant insertion. For the pilot-drill guided implant insertion, the times are itemized for short and long sleeves. The means, their standard deviations (SD), medians, minima and maxima are shown. The p-values are resulting from a pair-wise comparison. The p-values for statistically significant differences are marked in bold font

Group	Mean	± SD	Median	Minimum	Maximum	p-value
Pilot-drill guided	8.84 min	2.39 min	8.25 min	5.50 min	17.16 min	**< 0.001**
Full-guided	6.23 min	1.78 min	6.17 min	3.34 min	10.19 min	
Short sleeve	9.38 min	2.17 min	9.07 min	6.30 min	15.30 min	**0.041**
Long sleeve	8.41 min	2.50 min	8.10 min	5.50 min	17.16 min	

No statistically significant differences were found between gender, age, and a completed professional education prior to studying dentistry. A comparison considering the handedness was not performed as only one participant was left-handed.

## Discussion

### Comparison of accuracy between pilot-drill guided and full-guided implant insertion

In the presented examination, the accuracy of implant insertion using two different modes, either pilot-drill guided, or full-guided implant insertion, was evaluated in a model situation. When comparing the cumulated mismatch at the implant base and at the implant tip, it was obvious that the mismatch was higher at the implant tip. The mismatch was higher in the pilot-drill guided group for both areas. Similar results were found in a clinical study of Younes et al. where the apical global deviation was higher than the coronal global deviation [6]. Both deviations were higher in the pilot-drill guided group compared to the full-guided group, likewise. However, a statistically significant difference was not observed. Another clinical study revealed a statistically significant higher accuracy of the implant position at the coronal and apical aspect between all examined full-guided methods and the pilot-drill guided group [16], whereas Bencharit et al. could only find a statistically significant difference for the distal direction [17]. Considering the angulation between the planned and the achieved implant position, a higher accuracy was found for the full-guided compared to the pilot-drill guided method. This higher accuracy could also be observed in a clinical study where the angular deviation was higher in the pilot-drill guided group compared to the full-guided group: 5.95 ± 0.87° vs. 2.3 ± 0.92° [6]. However, this difference reached no statistically significant difference (*p* = 0.267). In other studies, the difference between the evaluated guided methods and the partially guided mode reached a statistical significance [16, 17]. This is in accordance with our findings where a statistically significant difference was found between the angulation *p* < 0.001. One reason for the higher accuracy of the full-guided method might be the closer guidance of the drills by the sleeve. Thus, the tolerance for changes of the position or angulation is reduced. Furthermore, by only guiding the pilot drill, changes of the implant position or implant angulation might occur. This can be advantageous when the implant position or angulation have to be corrected by experienced clinicians due to anatomical requirements. On the other hand, for inexperienced surgeons placing the fingers on adjacent teeth in order to stabilize their hand, this might lead to a pronounced deviation of the angulation [18]. Thus, in order to avoid this deviation, a closer guidance provided by full-guided templates could be beneficial for inexperienced surgeons. Considering the perforations of the buccal or lingual wall, a higher number was observed in the full-guided group. A reason for those perforations might be that the alveolar crest of the used mandibular model is rather thin. In those cases of a considerable narrow alveolar ridge, full-guided surgery might not practicable [6]. Already a minor mispositioning of the surgical guide can lead to a significant deviation of the implant without the possibility of corrections. Here, the use of a pilot-drill guided implant insertion might be favorable allowing a minor adjustment of the implant position and angulation.

### Comparison of accuracy between short and long sleeves for pilot-drill guided implant insertion

When comparing the accuracy achieved when using the long sleeves for the pilot drill with that using the short sleeve, it was obvious that long sleeves led to a higher accuracy. This finding is in accordance with Van Assche and Quirinen [19]. In their in vitro examination, it was stated that longer sleeves and longer drill keys led to a higher precision. This is to be explained by the tolerance that the drill must have in the drill key or sleeve to be able to rotate. Long sleeves will minimize this tolerance and thus lead to a higher accuracy compared with short sleeves. However, in some clinical situations, the application of short sleeves might be unavoidable due to a limited mouth opening [2].

### Time required to insert the implants

When comparing the required time for implant insertion, it was observed in our study that implant cavity preparation and insertion using the full-guided mode were statistically significantly faster than applying the half-guided mode. Apart from full-guided implant insertion providing a higher safety for implant position and angulation, in the pilot-drill guided mode, slight corrections of the position and the angulation are still possible after preparation of the pilot cavity [6]. In inexperienced surgeons, this might lead to a higher time consumption in order to check the position and angulation with the adjacent teeth. Furthermore, as the full-guided mode provides a depth stop, the surgeon does not constantly have to check the depth mark on the drill which might be not visible due to debris from the drilling.

### Individual factors influencing accuracy

The evaluated individual factors of the students which may potentially have influenced the accuracy of placing implants did not show any statistically significant differences. These findings are in agreement with a previous study examining an undergraduate population [15]. One reason might be the comparatively low number of participants in the current examination which would not reveal sufficient common demographic factors for analysis. Furthermore, it is possible that the manual skills of the dental undergraduates might be trained on such a level that there are no statistically significant differences when using the templates [20].

### Limitations of the study

The results of this study should be assessed with caution in that they are only applicable within the limitations of a laboratory set-up. The study was performed as an in vitro laboratory study using artificial mandibular models without a mucosa mask which is a limitation of the examination, as factors that may influence implant insertion clinically, such as soft tissue, bleeding, and saliva, are absent. Thus, the implant insertion in vitro has to be considered as easier than in a clinical situation [3]. However, a laboratory course can be considered as an essential part of the curriculum as it can prepare students for the clinical surgical intervention and minimize the risk for patients [10, 11]. Applying templates in such a setting is advantageous: first, it provides guidance for surgically inexperienced participants for implant position and angulation. Secondly, this setting allows a three-dimensional post-operative examination of the achieved implant positions enabling a direct feedback from the instructors to the participants regarding their accuracy. It therefore provides quality-controlled training which has shown a positive effect on patients’ safety [13]. This kind of three-dimensional radiographic examination might be difficult in a clinical setting with respect to the Radiation Protection Ordinances [21, 22]. Another limitation of the study might be the application of thermoformed templates. Compared to plotted templates, thermoformed templates have a reduced rigidity [1]. This might lead to a reduced accuracy in the achieved implant position when compared to the planned position. Considering the torsional stiffness and the resilience, plotted templates are superior to thermoformed templates. In our study, thermoformed templates were used as they could easily be produced in the department’s dental technician laboratory. Furthermore, the areas planned for implant insertion were on the left site tooth-bordered and on the right site adjacent to a tooth. Thus, adequate support of the template could be assumed. Additionally, it was questioned whether the differences between both fabrication methods might be of clinical impact as the differences found were in the tenth-of-a-millimeter range [1]. Another factor influencing the accuracy of surgical templates in clinical situations is number of teeth supporting them [23–25]. The accuracy in tooth-bordered gaps was found to be higher than in free-end gaps. One reason for the slightly higher mesio-distal mismatch for the full-guided implant insertion could be the lack of a distal tooth support on the right side of the mandibular model. Thus, a higher mobility of the template might be possible compared to the tooth-bordered gap on the left side of the model. A randomized distribution of both guiding modes between position 35 and 45 would have been a possibility to overcome this drawback which is a limitation of the study. In order to configure the study design for the participants as simply as possible, a randomization was not applied. Thus, the free-end gap in region 45 might have had an influence on the accuracy of the achieved implant position. However, a statistically significant difference in a clinical setting could not be found [23].

When considering the time required for full-guided implant insertion, it was found to be statistically significantly shorter compared to the pilot-drill guided. The three-dimensional planning for a full-guided implant insertion might require a longer time than the free-hand or the pilot-drill guided implant insertion. When counting the total time required, this amount of time has to be taken into account. This time consumption for the planning process was not assessed in the present study which is another limitation. However, it was not performed by the participating students but by an experienced surgeon so that there would be no comparable data. This topic should be examined in further studies.

## Conclusion

Within the limits of this laboratory study, it could be shown that the use of full-guided implant insertion led to a statistically significantly higher accuracy in a group of inexperienced surgeons. The hypothesis of the study that the full-guided implant insertion leads to a higher accuracy compared to pilot-drill guided implant insertion in inexperienced surgeons can be considered as fulfilled. Furthermore, a benefit regarding the time required for implant insertion could be observed for the full-guided mode. The impact of these finding on clinical situations has to be the subject of further studies.

## Abbreviations

CBCT: Cone beam computed tomography
DICOM: Digital imaging and communications in medicine
kV: Kilovolt
mA: Milliampere
mm: Millimeter
